# An Intertwined Network of Regulation Controls Membrane Permeability Including Drug Influx and Efflux in *Enterobacteriaceae*

**DOI:** 10.3390/microorganisms8060833

**Published:** 2020-06-01

**Authors:** Aurélie Ferrand, Julia Vergalli, Jean-Marie Pagès, Anne Davin-Regli

**Affiliations:** UMR_MD1, U-1261, Aix-Marseille University, INSERM, SSA, IRBA, MCT, Faculté de Pharmacie, 27 Bd Jean Moulin, 13385 Marseille CEDEX 05, France; ferrand.aurelie@hotmail.fr (A.F.); julia.vergalli@univ-amu.fr (J.V.); jean-marie.pages@univ-amu.fr (J.-M.P.)

**Keywords:** antibiotic resistance, *Enterobacteriaceae*, envelope permeability, efflux pump, ESR, global and local regulators, membrane protein, porins, TCS

## Abstract

The transport of small molecules across membranes is a pivotal step for controlling the drug concentration into the bacterial cell and it efficiently contributes to the antibiotic susceptibility in *Enterobacteriaceae*. Two types of membrane transports, passive and active, usually represented by porins and efflux pumps, are involved in this process. Importantly, the expression of these transporters and channels are modulated by an armamentarium of tangled regulatory systems. Among them, Helix-turn-Helix (HTH) family regulators (including the AraC/XylS family) and the two-component systems (TCS) play a key role in bacterial adaptation to environmental stresses and can manage a decrease of porin expression associated with an increase of efflux transporters expression. In the present review, we highlight some recent genetic and functional studies that have substantially contributed to our better understanding of the sophisticated mechanisms controlling the transport of small solutes (antibiotics) across the membrane of *Enterobacteriaceae*. This information is discussed, taking into account the worrying context of clinical antibiotic resistance and fitness of bacterial pathogens. The localization and relevance of mutations identified in the respective regulation cascades in clinical resistant strains are discussed. The possible way to bypass the membrane/transport barriers is described in the perspective of developing new therapeutic targets to combat bacterial resistance.

## 1. Introduction

During the last decades, the antibiotic resistance of Gram-negative pathogens has become particularly worrying due to the spreading of multidrug resistant strains that create clinical therapeutic outcomes [1,2,3]. In Gram-negative bacteria, such as *Enterobacteriaceae*, the cell envelope is a complex structure comprising two membranes delineating a periplasmic space representing the second liquid compartment of bacterial cell [4]. Protecting the cell from external medium, the outer membrane (OM) is an asymmetric bilayer of lipopolysaccharides and phospholipids, which contains non-selective porins, other substrate-specific protein channels embedded herein, and proteins involved in the membrane architecture, such as the lipoprotein or OmpA [5,6,7]. The periplasm and their various components, e.g., periplasmic binding proteins and peptidoglycan, are confined between the OM and the cytoplasmic or inner membrane (IM). IM is a phospholipidic bilayer involved in diverse physiological and metabolic functions, e.g., the transport of nutrients, energy production, signal transmission, etc. [8]. The IM also contains multidrug efflux pumps that actively expel toxic molecules and peptides from the bacterial cell [9].

These two membranes form a well-designed permeability barrier that efficiently act to control the intracellular accumulation of antibiotics and represent a prominent factors promoting intrinsic resistance of Gram-negative bacteria to a broad range of antimicrobial agents [6,9,10,11]. Interestingly, two main transport systems are located inside the two bacterial membranes: porins and passive transports of nutrients, sugars, are located in OM; while several important active transports occur in IM, energized by the membrane-associated proton motive force or ATP hydrolysis, including efflux pumps and various permeases involved in sugars, amino-acids, Lipo poly Saccharide (LPS), and peptide transport, etc. [6,8,9,12,13]. These transporters are also different from a structural point of view. Regarding porin structural and functional organization, a recent review has strongly summarized these aspects [10]. Briefly, the β-barrel organization in monomer subunits is described in general and specific porins, and the various orthologs present in *Escherichia*, *Enterobacter*, *Klebsiella* spp., exhibit large similarities in identity and function [6]. An important behavior of these porins is their involvement in the passive entry of small charged molecules as antibiotics across the OM [10].

**Figure 1:**
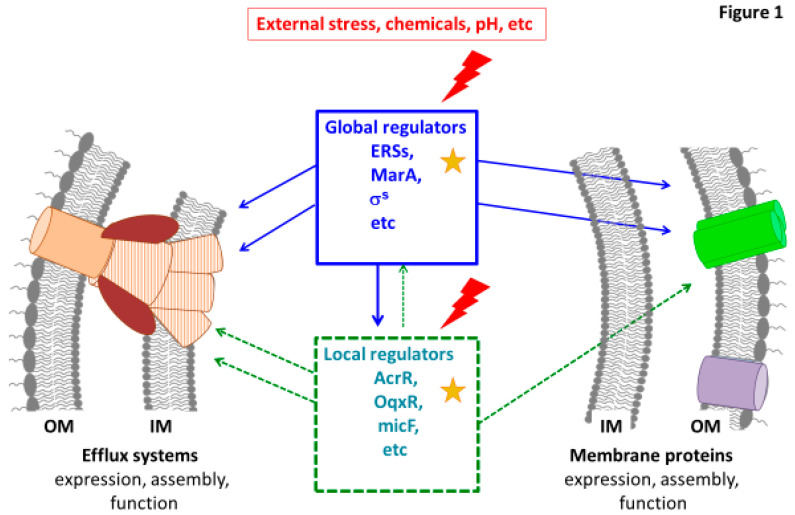
Illustration of the membrane permeability regulation. Some examples of global and local regulators are presented. These regulators triggered by environmental stresses control the key factors of membrane permeability: Efflux systems (in brown) and membrane composition including porins (in green). Some connections between global and local effectors are illustrated. The red lightning and yellow stars indicate possible environmental stresses and mutations occurring in genes encoding regulators identified in clinical resistant strains, respectively. OM: outer membrane; IM: inner membrane.

Regarding the drug transporters, multidrug efflux pumps encoded on the bacterial genomes belong to the ABC, MFS, SMR, MATE, PACE, and RND (super)families. Except for the ABC transporters, which use the ATP as an energy source to transport the drugs across the membrane, the other described multidrug efflux pumps are H^+^(or Na^+^)/drug antiporters [14]. These transporters carry drugs from the cytoplasm or the inner leaflet of the IM to the periplasm or to external medium with tripartite systems that comprise an inner membrane transporter (ABC, MFS, or RND), a periplasmic adaptor protein (PAP, previously known as the MFP for Membrane Fusion Protein), and an outer membrane channel (OMF, Outer Membrane Factor) [14,15]. The RND family contributes to the major membrane-associated mechanisms of efflux in *Enterobacteriaceae* and the more relevant complex described in clinical isolate belong to AcrAB-TolC system [16,17]. Importantly, due to their role in the bacterial adaptation to external stresses, these passive and active transports are tightly regulated in *Enterobacteriaceae*. It is mandatory to point out some key regulators present in different regulation cascades that can efficiently coordinate/balance the respective expression of passive diffusion and active expel of toxic compounds (Figure 1). The aim of this review is to discuss the key role of global transcriptional regulators including the XylS/AraC family members (Mar, Sox, Rob, Ram), and major Environmental Stress Response (ESR) systems, including two-component systems (TCS), in the emergence/spreading of membrane-associated mechanisms of resistance (MAMR) in clinical strains.

## 2. Regulators Type 1—Helix-Turn-Helix (HTH) Family Regulators, Including the AraC/XylS Family

Several regulatory systems have been involved in the development of multiple drug resistance and both structural and genetic investigations helped to understand and decipher their interactions [6,9,10]. Among them, *marA*, *ramA*, and *soxS* are efficient activators triggering the expression AcrAB-TolC tripartite efflux pump systems [18,19,20,21].

The control of membrane permeability is carried out by several ways at global or local levels (Figure 1):—positive regulation by general or specific transcriptional activators coordinating the expression of several genes,—negative regulation by repressors of activators or efflux pumps components.

### 2.1. Positive Regulation by Global or Local Transcriptional Activators 

The chromosomal transcriptional regulators of bacterial influx and efflux genes described in *Enterobacteriaceae* belong to AraC-XylS, MarR, and TetR families. All possess α-helix-turn-α-helix (HTH) DNA-binding motifs.

#### 2.1.1. The AraC-XylS Family

The family consists in more than 1500 proteins regulating cellular processes [22,23]. Most of them self-dimerize to function and possesses a conserved DNA binding domain with two HTH domains. They recognize similar DNA sequences in the promoter region of more than 60 regulatory targets (adaptation to the environment and protection against external aggressions) and are co-regulated between themselves through transcriptional cross talk [24].

##### The Mar Regulon

The *mar* (multiple antibiotic resistance) locus is an operon encoding for the key regulator MarA, which is reported among most of the *Enterobacteriaceae* [25,26]. MarR, its specific repressor naturally bounds on *marO* promoter and constitutively represses it. Moreover, AcrR, the repressor of the operon AcrRAB, can act negatively on *mar* regulon, by binding on *marO* [27]. The expression (or de-repression) of *marRAB* is the consequence either of (i) mutations in the MarR binding sites (reported in *Escherichia coli*), (ii) modification of MarR at the protein level preventing its repressor function, (iii) ligands binding MarR decreasing its affinity for the DNA, (iv) direct action of inductors of the system, and (v) specific feed-back dependent of AcrA or AcrB proteins concentration [28,29]. However, Multi drug resistant (MDR) clinical strains with MarR mutations were weakly reported [30]. Regarding post-translational level, the Lon protease is involved in the degradation of MarA and point mutations were detected in clinical strains [31]. 

MarA activates the Acr operon by binding to the intergenic region between *acrR* and *acrA*, lifting the repressive action caused by the AcrR repressor protein [32]. Moreover, in addition to modulating efflux, MarA up regulates expression of genes involved in membrane lipid trafficking and DNA repair, thus reducing tetracycline entry and quinolones DNA damages [33]. The study of Chetri et al. [34] have reported carbapenem-resistant *E. coli* isolates with overexpression of AcrAB-TolC and a down regulation of OmpF due to regulation by MarA [34]. The same work mentions that a treatment of these isolates with increasing concentrations of meropenem induces an increase of MarA and Rob expression and consequently an overexpression of AcrAB-TolC leading to a carbapenems resistance phenotype.

##### The Oxidative Stress Regulon SoxRS

SoxS is the effector of the *soxRS* global superoxide response regulon showing about 50% homology with MarA [35]. It is repressed by reduced SoxR or by AcrR and activated by superoxide-generating agents via conversion of SoxR (from reduced to oxidized state), its divergently transcribed local transcriptional activator [36]. SoxS is specifically down regulated by the sRNA MgR [37]. SoxS is involved in activation of the MDR phenotype by upregulating *acrAB* or activating *marA* and the phenotype induced by SoxS is similar to that induced by MarA [38]. Besides, *soxS* is upregulated in Δ*acrAB* mutants [28]. Mutations in *soxR*, inactivating its repressor function, have been identified in clinical isolates of *E. coli*, *Salmonella enterica*, *Klebsiella pneumoniae* from patients undergoing quinolones treatment [18,39]. In absence of *ramA*, mutations in SoxR are effective in particular after fluoroquinolones exposure and SoxS becomes the prominent regulator of the MDR in *Salmonella* sp. [18]. SoxS interfere with fluoroquinolones resistance mutations apparition in *E. coli*, by facilitating adaptation of cells and protecting them from the ions superoxides produced by bactericidal antibiotics [40]. 

##### The Rob Regulon

It regulates genes involved in resistance to antibiotics, organic solvents, and heavy metals [41]. Over-expression of Rob in *E. coli* or in *Erwinia* produces both increased organic solvent tolerance and low-level resistance to multiple antimicrobial agents, due to a two-fold increase expression of AcrAB [42,43]. There is an overlapping of the genes targeted by Rob and those under the control of Mar and Sox but Rob has a limited effect on the expression level [44]. Rob activates the target genes only after the binding of inducers such as medium-chain fatty acids and bile salts, with its C-terminal end. Its constitutive expression should maintain a basal low level MDR phenotype [45]. 

##### The Regulator RamA

RamA shares 45% identity with MarA and recognizes an overlapping set of targeted sequences [46]. RamA is described in *Enterobacter*, *Salmonella*, and *Klebsiella*, but absent in *Escherichia* in contrast to the MarA [47,48]. RamA elicited a high-level resistance to diverse antibiotics (chloramphenicol, tetracycline, tigecycline, fluoroquinolones, trimethoprim, etc.). It is responsible of the *acrA*-overexpression, in absence or reduced production of *marA* or *soxS* [49,50]. RamA is considered as the main global regulator of AcrAB and TolC overproduction, excepted in *E. coli* [50,51,52]. 

Considering the regulation, MarA could regulate the transcription of *ramA*; however, the constitutive expression of RamA results in a MDR phenotype even in the absence of the *mar* locus. RamA is a transcriptional activator of the Mar regulon and is also a self-governing activator of the MDR cascade [47]. RamR is the specific repressor of *ramA* expression and mutations responsible of MDR phenotype have been found in *Enterobacter* and *Salmonella* [52]. In carbapenem-resistant *K. pneumonia* isolates, mutations, insertion or deletion associated to frameshift and premature stop codon were identified in *ramR* genes [53,54]. Moreover, RamA is post-translationally regulated by Lon protease that degrades RamA or MarA [55,56]. Finally, its level is increased by mutational inactivation of *acrAB* expression or when efflux is inhibited, by feedbacks effects [57,58]. RamA plays a role in the oxidative stress response in partnership with SoxRS, and seems more important than MarA and SoxS in the development of MDR, except in *E. coli* [18,51,59]. It was expressed with higher levels in *Enterobacter cloacae*, *Klebsiella aerogenes*, and *Salmonella* MDR strains compared to MarA and was found to be more effective than MarA, RarA, SoxS for decreasing envelope permeability in *Klebsiella* [24,52,60]. RamA overproduction enhanced beta-lactamases-mediated resistance through activation of efflux pumps production [60]. Indeed, overexpression of RamA in carbapenem resistant isolates of *E. cloacae*, *K. aerogenes* induces a decrease of OmpF and OmpC expression level and an increase of the AcrAB-TolC expression leading to an extensively drug resistance phenotype [24,52]. 

##### The Regulator RarA

Besides *acrRAB*, the chromosome of *K. pneumoniae* contains another recently characterized locus, *rarA-oqxABR*, encoding an efflux system together with its regulatory elements [61]. The pump OqxAB is associated with resistance to quinoxalines, quinolones, tigecycline, nitrofurantoine, chloramphenicol [62]. RarA is an AraC-type transcriptional regulator that is overproduced as OqxAB when the negative regulator OqxR does not work [51,63,64].

*rarA* overexpression resulted in the differential expression of 66 genes (42 upregulated and 24 down regulated) [65]. Under the clusters of orthologous groups functional classification, the majority of affected genes belonged to the family of cell envelope biogenesis and post-translational modification, along with genes encoding previously uncharacterized transport proteins and the porin OmpK35 [65]. RarA is active in *Klebsiella, Enterobacter,* and *Serratia,* and is proposed to be the most effective positive effector of the *Klebsiella* MDR after RamA [62].

##### TetD and NimR (YeaM)

TetD recognizes the same sites than MarA and share 43% identity with it. It is encoded by transposon *Tn*10. Its expression is negatively regulated by TetC [66]. It is associated to resistance to tetracycline but its expression is not induced by this drug. Mutations of its promoter have been described, however its physiological function is unknown yet. NimR is the transcriptional activator of the efflux pump of the MFS family, NimT (for Nitroimidazole transporter) responsible of the 2 Nitroimidazole resistance in *E. coli* [67].

#### 2.1.2. Other Regulators

H-NS (histone-like structuring nucleoid protein) regulates the expression of porins and several efflux pumps in *E. coli*, *S. Typhimurium*, and *K. aerogenes* in response to osmotic stress [68,69]. There is evidence that this H-NS also controls expression of OmpX [70]. SdiA, a protein that controls cell division genes in a dependent-manner upon quorum sensing positively regulates the AcrAB expression [71]. FIS is a nucleoid-associated global regulatory protein that modifies *acrAB* transcriptional activity in response to various growth conditions and can also bind to a site within *marO* upstream the marbox. This regulator is proposed to limit the overall level of negative superhelicity and stabilize the local DNA architecture of certain promoters, providing an additional two-fold stimulation to MarA-, SoxS-, and Rob-mediated activation of transcription [72]. CsrA is a RNA binding protein able to bind to the 5’ end of the transcript encoding AcrAB and is responsible of stabilization of the mRNA. Using a feedback, inactivation of CsrA increases *ramA* expression due to a AcrAB down expression [57]. Finally, cellular metabolites from enterobactin, cysteine, and purine biosynthesis, gluconeogenesis are able to activate (i) SoxS by inactivating SoxR (ii) and MarA by inactivating MarR [28].

### 2.2. Repressors of Pump Genes Expression

#### 2.2.1. TetR/AcrR Repressor Family

It is the more abundant family of regulators of MDR, antibiotics biosynthesis and pathogenesis processes [73]. AcrR was identified as repressor of AcrAB, MarAB, SoxRS [27]. Many operons encoding efflux pump components contain an associated regulatory gene that plays a key specific role in controlling the expression of the corresponding pump. AcrR represses both its own and *acrAB* transcription [74]. Mutations in *acrR* have been shown to de-repress *acrB*. Such mutations have been found in clinical isolates of *E. coli*, *S. Typhimurium*, *K. aerogenes,* and *K. pneumoniae* [75,76,77]. 

RamR is the repressor of RamA and several mutations were identified in its dimerization domain, resulting in a decrease of DNA-binding affinity on the promoter of *ramA* in *Salmonella* [78]. It recognizes multiple chemicals such as bile components, crystal violet, ethidium bromide, rhodamine G, and the resulting association reduces its proper affinity for DNA target sites [79]. Mutations and deletions were also identified in clinical strains of *K. pneumoniae* or *aerogenes* and of *E. cloacae* and were considered as a serious prevalent mechanism associated to tigecycline resistance [31,52,80]. 

#### 2.2.2. GntR Repressor Type: OqxR

The GntR family of transcription factors (TFs) is a large group of proteins present in diverse bacteria and regulating various biological processes. Proteins are comprised of a DNA-binding domain and a signaling domain, linked together. Among them, in *K. pneumoniae,* OqxR down regulates *rarA* and *oqxAB* expression [80,81]. It is located neighboring to *oqxAB* [62]. In clinical *K. pneumoniae* strains, mutations and frameshift deletions were identified associated to RamR mutations and were responsible to tigecycline resistance [61,64,80,82].

## 3. Regulators Type 2—TCS and Other Regulators

Detection of environmental change is the first adaptation/defense line of bacteria face to detrimental hazards to its basic physiological activities. Gram-negative bacteria have developed several systems that are able to sense the external medium modifications such as pH, osmotic strength, oxidative stress, nutrient starvation, toxic chemicals, and transfer the corresponding signal via transduction to appropriate internal regulators [83,84,85,86,87,88,89,90,91,92,93]. These regulators can directly or indirectly (regulation cascades) manage the expression of various components to adapt the bacterial membrane structure and composition allowing the bacteria to ensure their growth and survival [94,95]. 

### 3.1. Envelope Stress Response (ESR) and TCS

Among them, two-component systems (TCS) represent a worldwide process used to respond to external stimuli and transduce the signals across membranes [96]. TCS comprise a sensor protein that is located outside the IM and an associated response regulator located in the cytoplasm. Briefly, in response to external stimuli, the receptor domain of the sensor protein induces the autophosphorylation of its histidine residue through adenosine triphosphate hydrolysis. The phosphate is then transferred to the response regulator protein allowing it to target DNA and modulate gene expression [97].

The input and output domains of TCS (receptor domain of sensor protein and domain targeting DNA of the response regulator protein, respectively) being variable, TCS are able to recognize a variety of molecules and regulate a plethora of genes [97]. Additionally, multiple TCS may be active in the same bacterial cell [24,85,89,98]. 

In *E. coli* and closely related *Enterobacteriaceae*, the Envelope Stress Responses (ESRs) involve different complex regulator cascades and proteins. The stress-responsive alternative sigma factor (σ^E^), the phage shock protein (Psp) and the TCS CpxAR, Rcs, BaeSR, PhoPQ, and EnvZ/OmpR regulate processes in response to many signals of envelope defects, such as OM and periplasmic misfolded proteins, IM alteration, disruption of proton motive force, perturbated Tat system, LPS, and Peptidoglycan (PG) defects [93,99,100,101,102]. It should be note that even in the absence of stress, the biogenesis of the cell envelope require continuous regulation [99,101]. These regulators are considered as the ‘watchdogs of the envelope’, and CpxAR and σ^E^ are usually considered as the main ESR systems active in *E. coli* [85,93]. There is convincing evidence that these systems, especially Cpx, can sense and respond to cell wall damage, while the molecular signals remain mostly unknown in clinical isolates [100,101]. 

### 3.2. ESR, TCS, and Antibiotic Resistance 

Overall, antibiotics target physiologic functions of bacteria inducing the regulatory network to challenge the antibiotic attack, highlighting the tight connection between antibiotic resistance and the maintenance of metabolic homeostasis. Previous transcriptomic studies have reported that β-lactams lead to the expression of genes controlled by ESR systems [103]. Interestingly, the authors highlight the fact that β-lactams antibacterial activity is bolstered by the induction of a toxic malfunctioning of the overall targeted machinery [103]. Several TCS have been characterized to be involved in antibiotic resistance phenotype and can play a direct role in modulating the antibiotic concentration in bacterial cell [97,104]. 

#### 3.2.1. INFLUX Modulation

An important aspect of TCS involvement in bacterial permeability concerns their control in the porin expression including the balance of porin type, e.g., OmpC versus OmpF or other enterobacterial ortholog porins, and its level of expression [10,105,106,107]. For instance, resistance to β-lactams in an isolate of *Salmonella enterica* serovar Typhimurium has been mainly attributed to the decrease of porin mRNA induced by Cpx [106]. TCS also induce antibiotic resistance in *E. coli* and *K. pneumoniae* via a modification of porin expression [97,108]. TCS, such as PhoPQ, PmrAB, and Rcs also induce resistance to antibiotics by the modification of the cell surface [97,109]. It must be noted that PhoP-PhoQ and mutations in genes encoding proteins playing a role in LPS expression can also modulate the functional assembly of porins [99,110]. 

Moreover, other ESRs such as σ^E^ can also modulate the expression of genes directly involved in the control of membrane proteins expression [90,111]. This regulation lead to decrease influx and consequently the susceptibility to numerous antibiotics such as β-lactams, aminoglycosides, polymyxin B, colistin, and fluoroquinolones [97,104,105,106].

Mutations of regulatory components have been found to confer drug resistance in clinical isolates of *Enterobacteriaceae*: regarding TCS, mutations in *pmrAB* in *K. pneumoniae* and *S. enterica* and in *phoPQ* in *K. pneumoniae* and *E. coli* induce resistance to colistin [20,104]. A mutation in *baeS* has been found to be associated with a decrease susceptibility to monobactam, aztreonam, and ceftazidime in *E. coli* and *K. pneumoniae* [112].

Regarding CpxAR, recent studies enlighten functional characterization of the first gain-of-function mutation of CpxA, which was identified in a multidrug resistant clinical strain of *K. aerogenes* [113]. This mutation in this TCS regulator system confers resistance to β-lactams and aminoglycosides by regulating the expression of porin genes involved in their uptake (*omp35*, *omp36*) or extrusion (*acrD*) [94]. Together with other previous studies, these data document the role of the Cpx system in antibiotic resistance in laboratory and clinical strains of enterobacteria [91,101,105,106,112].

#### 3.2.2. EFFLUX Modulation

In addition to the effect on antimicrobial influx, regulatory systems also lead to modulate the expression level of efflux transporters; which often represents one of the first step to increase the level of resistance [104,106]. While the primary regulators of efflux pumps in bacteria are known to be the members of XylS/AraC family (see above), TCS are also involved in the regulation of efflux transporters. For instance, the down-regulation of OmpD conjointly to the AcrD up-expression by BaeSR and CpxAR in a *S. enterica* isolate has been associated with the ceftriaxone resistant phenotype [105]. Similarly, the resistance to aminoglycoside of a *S. enterica* isolate is associated with an increase level of mRNA of *acrD* and *mdtA* induced by CpxR [106]. 

#### 3.3. sRNAs

The first study of a post-transcriptional repression of porin expression by the small non-coding regulatory RNA (sRNA) MicF has been published in 1984 [for recent reviews see [85,86]. This sRNA is located closed to the ompC gene and prevent the *ompF* mRNA translation by direct base-pairing to a region corresponding to the ribosome binding site and the start codon [85]. During the last decades, several sRNAs have been identified and characterized, and their key role in the control of bacterial membrane permeability is now recognized [85,88,90]. Numerous sRNAs are involved in regulating the porins expression such MicF, MicC, MicA, RygB, RseX, CyaR, or transporters expression such as RydC, DsrA, and RyeB. Their involvement in drug resistances associated to uptake and efflux is reviewed in the work of Felden, et al. [88]. 

**Figure 2:**
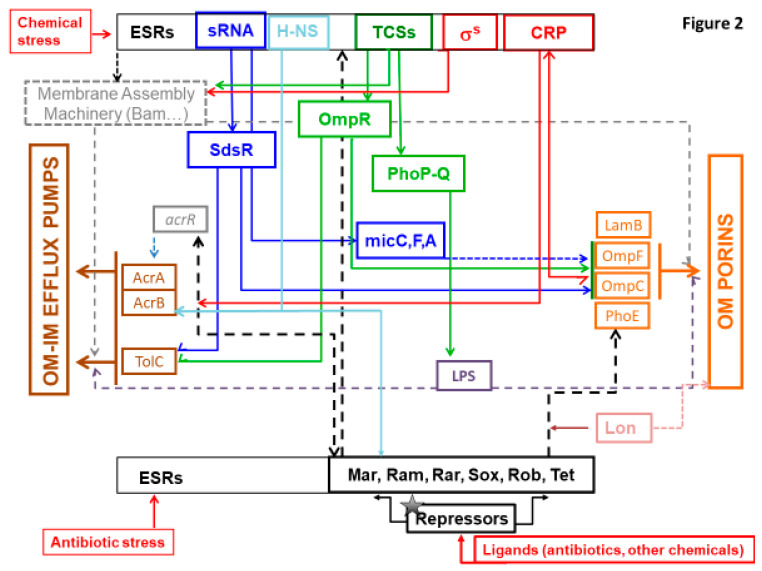
Schematic representation of several bacterial effectors that control membrane permeability and the In and Out of molecules. The various regulation cascades that modulate the OM permeability including porins (orange square, on the right of the diagram), and the expression of major efflux pumps (AcrAB, OqxAB; brown square, on the left of the diagram) are illustrated. In response to external stimuli (red arrows), such as chemicals including antibiotics, Envelope Stress Response (ESR) effectors are triggered and activate multiple regulation pathways, promoting the development of MDR phenotypes in *Enterobacteriaceae*. Among them, the major type 1 regulators including AraC-XylS family members and Tet are shown (at the bottom of the diagram) with their corresponding repressors (in black). MarA, RamA (absent in *E. coli*), RarA, SoxS, and Rob activate the AcrAB-TolC expression (and OqxAB in *K. pneumoniae*). They also modulate the porin expression and are involved in the membrane lipid trafficking. The Lon protease (in pink) degrades RamA and MarA. Two-component systems (TCS) (in green, at the top of the diagram) are among the main ESR effectors of type 2 regulators. They regulate processes in response to chemicals and other signals of envelope defects and subsequently controls the membrane machinery (grey square), LPS integrity (purple square), and porins expression. TCS, such as PhoPQ, induce antibiotic resistance by controlling the modification of LPS and the assembly of porins. The TCS regulator OmpR (in green) modulate the expression of the major porins under an osmotic stress, promoting a resistance to B-lactams. The H-NS (in light blue) is also involved in the response to osmotic stress and regulates the expression of efflux pumps and porins in *E. coli* and *K. aerogenes*. The Sigma factor (in red) regulates genes involved in expression of membrane protein. The sRNAs (in blue) regulate (and are regulated by) the ESRs. They have multiple mRNA target and allow to mediate a rapid and efficient response. sRNAs such as MicF, MicC, MicA (in blue) are involved in the regulation of porins expression. sRNAs also mediate the regulation of efflux pump expression with, for instance, the binding of SdsR (in blue) to the TolC mRNA. The Cyclic AMP Receptor Protein (CRP, in red), one of the most important TF in *E. coli*, is also involved in the regulation of efflux systems and porins expression. Overall, ESR effectors regulate LPS biogenesis that is involved in the OM proteins assembly and, consequently, the function of porins and TolC (grey dashed lines).

Noticeably, sRNAs have a substantial role in regulatory pathways as they regulate and are regulated by ESRs and allow a rapid and efficient response as recently summarized in excellent reviews detailing the mode of regulation and the respective targets [88,99,114,115,116,117]. This mechanism of regulation is also important for the balance of energy cost in bacterial cell: by blocking the translation of porins in initial step, it allows substantial energy saving that are required during the production of proteins and their sophisticated assembly in bacterial membrane [6]. The involvement of some sRNA in the control of membrane permeability are illustrated in Figure 2.

### 3.4. Other Mechanisms/Partners 

It must be emphasized how the regulatory network is pleiotropic by (i) the large repertoire of signals triggering regulators, (ii) the substantial number of regulatory partners [89,118,119] acting at different levels (post-transcriptional, transcriptional, translational), in which most of them (iii) have multiple targets, and (iv) induce variable responses [83,110]; and (v) the overlap of the pathways leading to cross regulation (CpxAR and EnvZ/OmpR, CpxAR and σE, CpxAR and BaeSR, CpxAR and *mar* operon, σ^E^ and PhoPQ, Rcs and PhoPQ, Rob and Mar etc.). The complexity of the regulatory network of a bacterium is much greater than what we know. It is necessary to keep in mind that, to this simplified scheme sketched here, other processes not mentioned in this work should be added. For instance, the regulation by RNA attenuators [90], protein activity sensing or moonlight proteins that carry out at least two different functions [120]), to which must be added the many signals and new players that research is progressively discovering.

New systems and effectors involved in regulatory pathways and, consequently, in antimicrobial resistance, are constantly being discovered as evidenced by the recent discovery of YciF involved in the ESR responding to bile salt stress (see below) [95], or the recently characterized ESR ZraP-SR, which is required to maintain envelope integrity against biocides, and is involved in antibiotic resistance [111]. Additionally, this latter work allows to identify 25 genes regulated by ZraR, although the authors suggested that other response effectors in the ZraSR pathway may be involved [111]. Indeed, several studies point out that many signals/regulators/effectors remain to be discovered [89,101,111,118].

### 3.5. Key External Factors Other Than Antibiotics

It is also interesting to mention that some drugs, currently used for human and animal treatments, are able to manipulate the expression of global and local regulators that contribute to membrane-associated mechanisms of resistance. Several reports have mentioned the effect of various chemicals, such as salicylate, triclosan, imipenem, pharmacological agents, and recently tylosin, biocides (chlorhexidine), that can favor/stimulate the emergence of mutations on regulators, or on the antibiotic susceptibility [24,98,121,122,123,124,125]. 

Remarkably, the bile stress has been recently described as powerful agent able to modulate the OM permeability including porin and efflux pump expression, via genetic control by CspE proteins in *Salmonella* [95,118]. These bacterial cold shock proteins acting as RNA chaperones are up-regulated by bile stress and can act together with new identified YciF in the regulation of porin and in the resistance against bile stress mediated by efflux pump [95]. 

Moreover, it is important to mention that the LPS synthesis and assembly [10,99,110] are also involved in the OM proteins assembly and subsequently can alter the function of OM channels including porins and TolC [10,126]. 

It worth to note that an environmental stress can induce DNA damages triggering an SOS response leading to activate a regulatory cascade (involving ESRs) for the repair of damages as well as the inhibition of cell division [92,127]. Thus, the SOS response induces a high genetic diversity notably with the incorporation of new mutations by the upregulated DNA polymerase or via horizontal transfer of resistance genes, promoting acquisition of new antibiotic resistances [90,98]. Another note in connection with the SOS response is that it promotes the formation of persisters [128]. 

## 4. Concluding Remarks 

Mutations occurring in global activators or all corresponding genes and negative repressors have been reported in various clinical strains [34,47]. We can mention the recent study of an isolate of *Salmonella* paratyphi A, resistant to macrolides, that exhibits mutations in numerous regulators (*marA*, *marR*, *E. coli soxS*, and *soxR*, *cpxA*, *msbA*, *baeR*, etc.) conferring multi-drug resistances [129]. Similarly, Majewsky et al., recently points on the expression of MarA, RamA, SoxS in MDR *E. cloacae* isolates that affect conjointly the porins and efflux pumps expressions [24]. A closed report described the absence of OmpK35 associated with the over expression of efflux pump in *K. pneumoniae* isolates carbapenem-resistant [130]. Interestingly, the colistin resistance phenotype reported in this study is likely due to the upregulation of the regulator RamA and SoxS that causes an overexpression of AcrAB-TolC efflux pump. In *E. coli*, strains isolated during faropenem treatment, mutations in MarR, OmpC and AcrB, leading to cross resistance have been described [131]. Same observations were made in carbapenem resistant *E. cloacae* after fluroquinolones contact [81]. 

This redundant use of mechanisms clearly supports the concern to approach the drug flux inside Gram-negative bacterial cell as a whole: we must take into account the two fluxes regulating the internal concentration, the Influx and the Efflux. For living cells, the control/management of membrane transport involved sophisticated and intertwined ways of regulation, which play a role at various levels, e.g., transcriptional, translational, and post translational. Moreover, we can note that the redundancy and accumulation of these "controllers" of membrane permeability and transporters is a key aspect allowing the bacterial cell to (i) detect/respond to the presence of noxious compounds in the medium and (ii) select an appropriate level of membrane permeation. This large redundancy of controls confers rapid fine-tuned responses, versatility, flexibility, and contributes to a limited cost for efficient adaptation by multiplying the available regulation target for a key function of bacterial metabolism the membrane transport [83,88,97,126,128,129,132]. 

At this moment, a large collection of regulators has been described in *Enterobacteriaceae*. Importantly, the molecular signal inducing their expression and their precise role in the physiology remain uncharacterized for some due to their redundancy and the entanglement of their respective actions on bacterial physiology and the resulting feedback. However, it is important to integrate all these various regulators and cascades in a global view of bacterial envelope physiology that must take into account the bacterial fitness and energy cost (see Figure 2). 

Figure 2 portrays this complex schema with the different actors /factors/regulators that can balance the entry/expel fluxes (Influx vs. Efflux). It is important to mention that in addition to antibiotics, several other molecules such as antimicrobial peptides, disinfectants, pesticides, detergents, etc., can trigger the bacterial response and induce MAMR [98,122,124,125]. This explains why ESR systems are involved or contribute in the sensing and transmission of internal signal that activate the genetic cascades controlling the envelope permeability and transports. Consequently, the TCS systems have been proposed as new target for original antibacterial agents in order to impair the bacterial adaptation and virulence [86,119,133,134,135]. In the same way, targeting sRNAs is currently being considered in view of their central implication in the regulatory network leading to antibiotic resistance [88,90]. An inhibitor of sRNA-Hfq (chaperone protein of sRNAs interaction with mRNA) interaction has been identified [136]. A recent paper describes the possibility to inhibit the trigger of antibiotic resistance by using LexA target to inhibit the SOS response involved in fluoroquinolone and drug persisters [137,138,139]. However, it must be noted that the redundancy of the regulatory network may imply that bacteria use a different pathway or acquire new mutations to hijack the effect of the drug [18], thus a better knowledge of the whole network is needed. 

Today, the molecular dissection of the permeability regulation is a prominent objective not only for preparing the fight against MDR bacteria but also to understand the genetic keys that have been selected during evolution by microorganisms face to environmental stresses. The availability of numerous genome sequences for several human pathogens associated to the development of recent methods for measuring antibiotic accumulation and antibacterial activity pave the ways for new targets and new strategies to combat antibiotic resistance in Gram-negative bacteria.
